# Role of IRF4 in resistance to immunomodulatory (IMid) compounds^®^ in Waldenström’s macroglobulinemia

**DOI:** 10.18632/oncotarget.22872

**Published:** 2017-12-04

**Authors:** Elisabeth Bertrand, Nathalie Jouy, Salomon Manier, Guillemette Fouquet, Stéphanie Guidez, Eileen Boyle, Stéphanie Noel, Cécile Tomowiak, Charles Herbaux, Susanna Schraen, Claude Preudhomme, Bruno Quesnel, Stéphanie Poulain, Xavier Leleu

**Affiliations:** ^1^ Univ. Lille, UMR-S 1172, Factors of Persistence of Leukemic Cells Team, JPARC - Centre de Recherche Jean-Pierre AUBERT, Neurosciences et Cancer, Lille, France; ^2^ Inserm, UMR-S 1172, Factors of Persistence of Leukemic Cells Team, Lille, France; ^3^ Service des Maladies du Sang, CHU, Lille, France; ^4^ Institut pour la Recherche sur le Cancer de Lille, Factors of Persistence of Leukemic Cells Team, Lille, France; ^5^ Laboratoire d’Hématologie, Centre de Biologie et Pathologie, CHU, Lille, France; ^6^ Service d’Hématologie et Thérapie Cellulaire, Hôpital La Milétrie, et Faculté de Médecine, CHU, Poitiers, France; ^7^ CIC Inserm 1402, CHU, Poitiers, France; ^8^ Plateau de Cytométrie, BioImaging Center Lille Nord de France, BICeL Campus Hospitalo-Universitaire, Lille, France

**Keywords:** Waldenström’s macroglobulinemia, IMid compounds, resistance, IRF4

## Abstract

**Background:**

Immunomodulatory drugs, IMid compounds, are active in Waldenström's macroglobulinemia (WM), although in a lesser extent than multiple myeloma, where it was initially developed. We hypothesized WM tumour cells might develop mechanisms of resistance, and sought to identify and describe these mechanisms.

**Material and Method:**

MM and WM-derived cell lines, and Waldenström's CD19+ cells were treated using both lenalidomide and pomalidomide. Stable CRBN expressing cells were generated.

**Results:**

WM-derived cells were resistant to IMid compounds. We demonstrated a modulation of the downstream targets of IRF4, despite low expression of cereblon, and hypothesized IRF4 was the cause for resistance to IMid compounds. We ruled out the role of various IRF4 regulatory mechanisms, and other pathways activating WM tumor cells, such as B cell activators.

**Conclusion:**

This study demonstrated that mechanisms of resistance to IMid compounds could be not related to cereblon. IRF4 was identified as the potential mechanism of resistance to lenalidomide and pomalidomide in WM. It potentially explains the lesser activity observed in the clinic in WM. Interestingly, some WM patients benefited strongly to lenalidomide and pomalidomide, and future studies will have to describe the indirect mechanisms of IMid compounds in WM, possibly related to an immune-mediated process.

## INTRODUCTION

Thalidomide and its derivatives, lenalidomide and pomalidomide, are immunomoludatory drugs, IMid compounds^®^, used with success in treatment of B cell malignancies such as multiple myeloma (MM) [1]. We recently conducted a phase 1/2 clinical trial of single agent lenalidomide, the first commercially available IMid compounds, in RRWM (Relapse Refractory WM), and interestingly, in patients where some drug activity was proven, the disease was controlled on a prolonged way. However, WM demonstrated response to lenalidomide to a lesser extent compared to what was published in myeloma overall, where it was initially developed [2]. We hypothesized WM tumour cells might develop mechanisms of resistance to lenalidomide, and possibly across IMid compounds, hampering its effects; and sought to identify and describe these mechanisms.

Waldenström's macroglobulinemia (WM) is a low grade B-cell lymphoproliferative lymphoma characterized by an accumulation of lymphoplasmacytic cells in the bone marrow and a monoclonal IgM secretion [3]. Despite progresses in disease management, cure is yet to be found with a median survival of 5 to 11 years after diagnosis of active WM [4]. Studies have put the light on a somatic mutation on MYD88, MYD88^L265P^, a molecular signature of most if not all WM patients, that was triggering the activation of the nuclear factor кB (NFкB) pathway [5, 6]. Toll-like receptors (TLRs) pathway, whose MYD88 is part of, is one of the main WM signaling pathways, along with and interconnected to B-cell Receptor (BCR) and Bruton's tyrosine kinase (BTK) pathways. All of these pathways signal through a regulation of Interferon Regulatory Factor 4 (IRF4) [7].

Studies have demonstrated that the effects of lenalidomide and pomalidomide on malignant cells involved binding to the protein cereblon [8], part of the E3 ubiquitin ligase complex. This binding led to a change of substrates ubiquitinylated by the complex and a proteosomal degradation of both zinc finger proteins Ikaros (IKZF-1) and Aiolos (IKZF-3)[9]. Ultimately, a down regulation of the interferon regulatory factor 4 (IRF4), a transcriptional factor with a central role in MM cells functioning is observed [10, 11]. It has been shown that a low level of expression of CRBN could induce a resistance to lenalidomide and pomalidomide, IMid compounds, in cells [8], however little is known regarding IKZF-1/3, c-Myc and IRF4.

Herein we try to identify and characterize the mechanism of resistance of WM cells to IMid compounds, lenalidomide and pomalidomide.

## RESULTS

### Immunomodulatory agents lenalidomide and pomalidomide treatment does not affect WM cell viability and apoptosis

**Figure 1:**
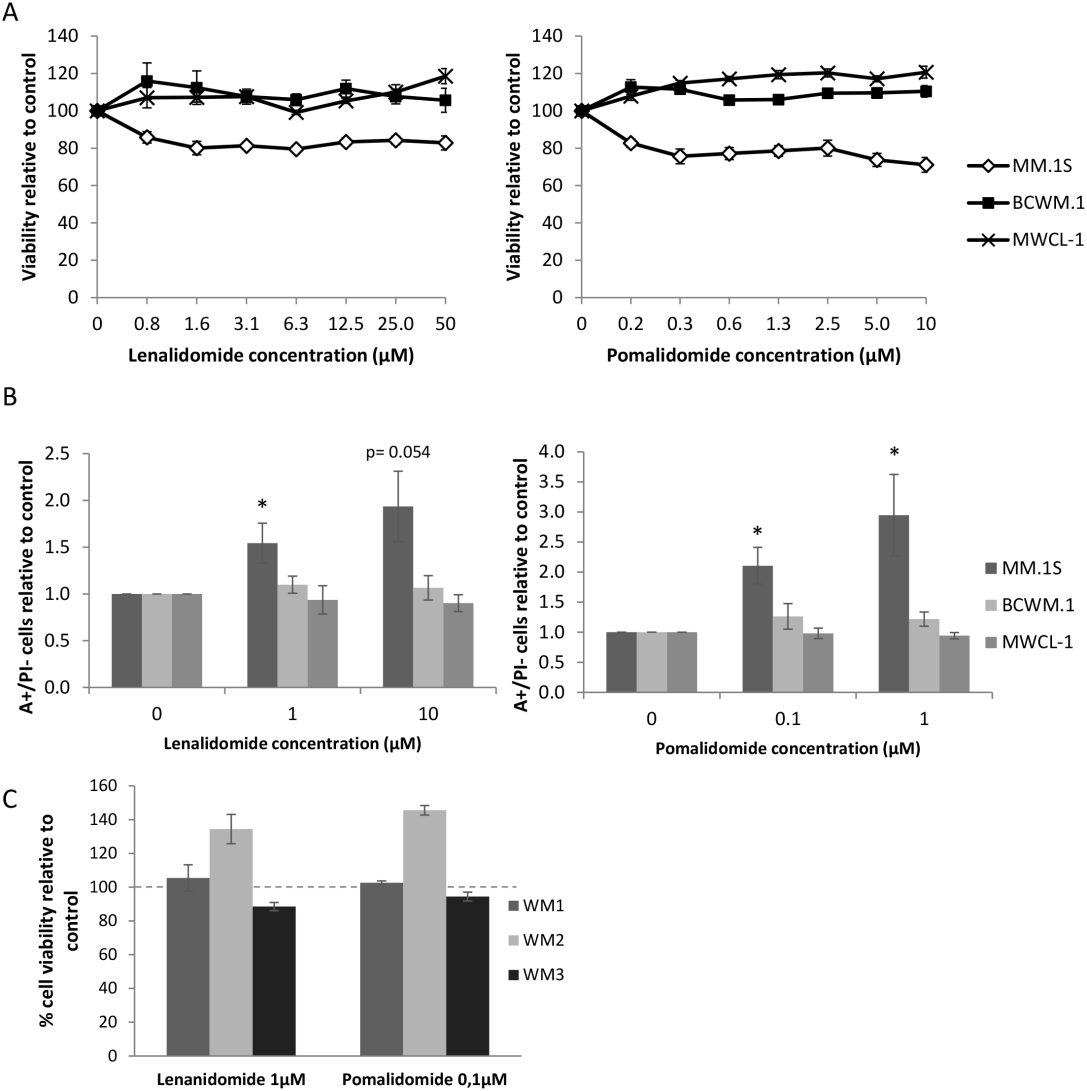
WM cell lines are not responsive to lenalidomide and pomalidomide treatments **(A)** Viability (MTS) of the MM or WM cell lines treated with lenalidomide (left) or pomalidomide (right) for 48 hours. Dots represent the mean of three different experiments +/- SD. **(B)** Quantity relative of annexin V positive and PI negative cells after 48 hours treatment of lenalidomide (left) or pomalidomide (right) at the indicated concentrations. Histograms represent the mean of six different experiments +/- SEM. ^*^: *p*<0.05. **(C)** Viability (MTS) of CD19 positive PBMCs isolated from three different WM patients treated with lenalidomide (1μM, left) or pomalidomide (0.1μM, right) for 48 hours. Error bars show standard deviation of triplicates.

Cell viability was assessed using MTS assays 48 hours after lenalidomide and pomalidomide, IMid compounds, treatment, at increased concentrations. As previously published [8], MM.1S cell line showed a decline of its viability in a dose- and time-dependent manner. BCWM.1 and MWCL-1 however were not responsive to either lenalidomide or pomalidomide (Figure 1A). The same results were obtained when treating for 72 hours and any other time point (data not shown).

We next investigated the effects of lenalidomide and pomalidomide treatment on apoptosis using an annexin V and propidium iodide staining. Similarly, apoptosis was induced in MM.1S cells after 48 hours of treatment whereas no significant difference was observed in WM cells (Figure 1B).

We then confirmed these results on CD19+ selected tumour cells from three WM patients (Figure 1C). No viability change was observed in CD19+ cells from two out of the three patients tested; CD19+ cells from the third patient were found to be partially responsive to lenalidomide and pomalidomide. Altogether, these results suggest that there is a mechanism of drug resistance in WM preventing lenalidomide and pomalidomide from having an effect on cell viability and apoptosis, unlike in MM.

### CRBN overexpression does not impact lenalidomide and pomalidomide sensitivity

**Figure 2:**
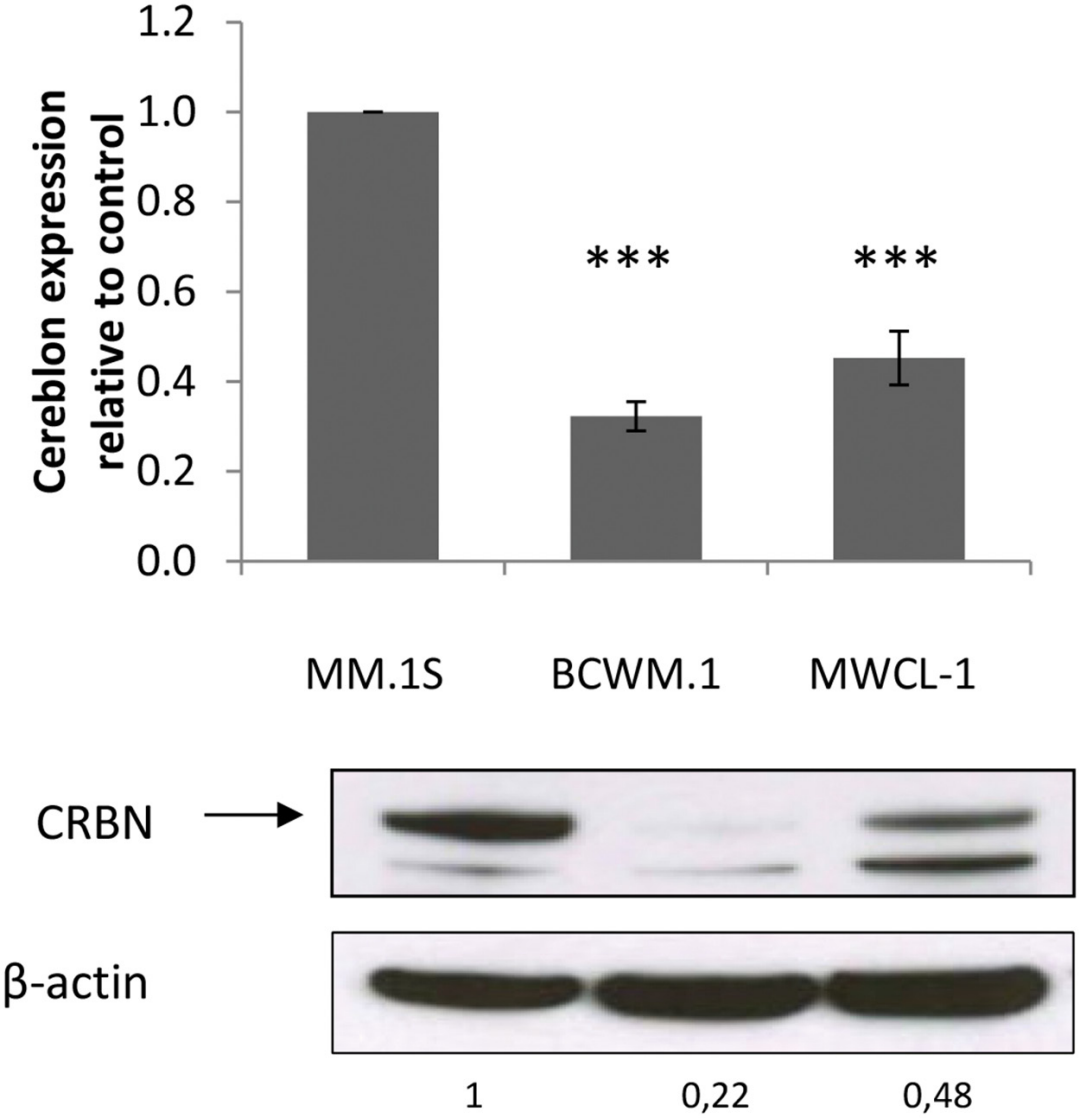
CRBN expression is lower in WM than in MM cells qRT-PCR analysis (top) shows that CRBN mRNA expression is lower in WM cell lines BCWM.1 and MWCL-1 compared to MM.1S. Western blot analysis shows a visible difference of CRBN expression in WM cell lines compared to MM.1S. Band intensities were quantified using ImageQuant TL software, normalized to their respective GAPDH bands and expressed comparatively to MM.1S. Histograms represent the mean of three different experiments +/- SD. ^***^: *p*<0.001

In order to promote their antitumor effect, lenalidomide and pomalidomide bind to the protein cereblon (CRBN) [12]. It has been shown that a lack of CRBN could induce a resistance to these drugs in multiple myeloma [8] and B cell lymphoma [13]. We thus investigated whether WM cells were expressing CRBN. The results showed that both mRNA and protein levels were much lower in the WM cell lines than in MM.1S cells (Figure 2).

**Figure 3:**
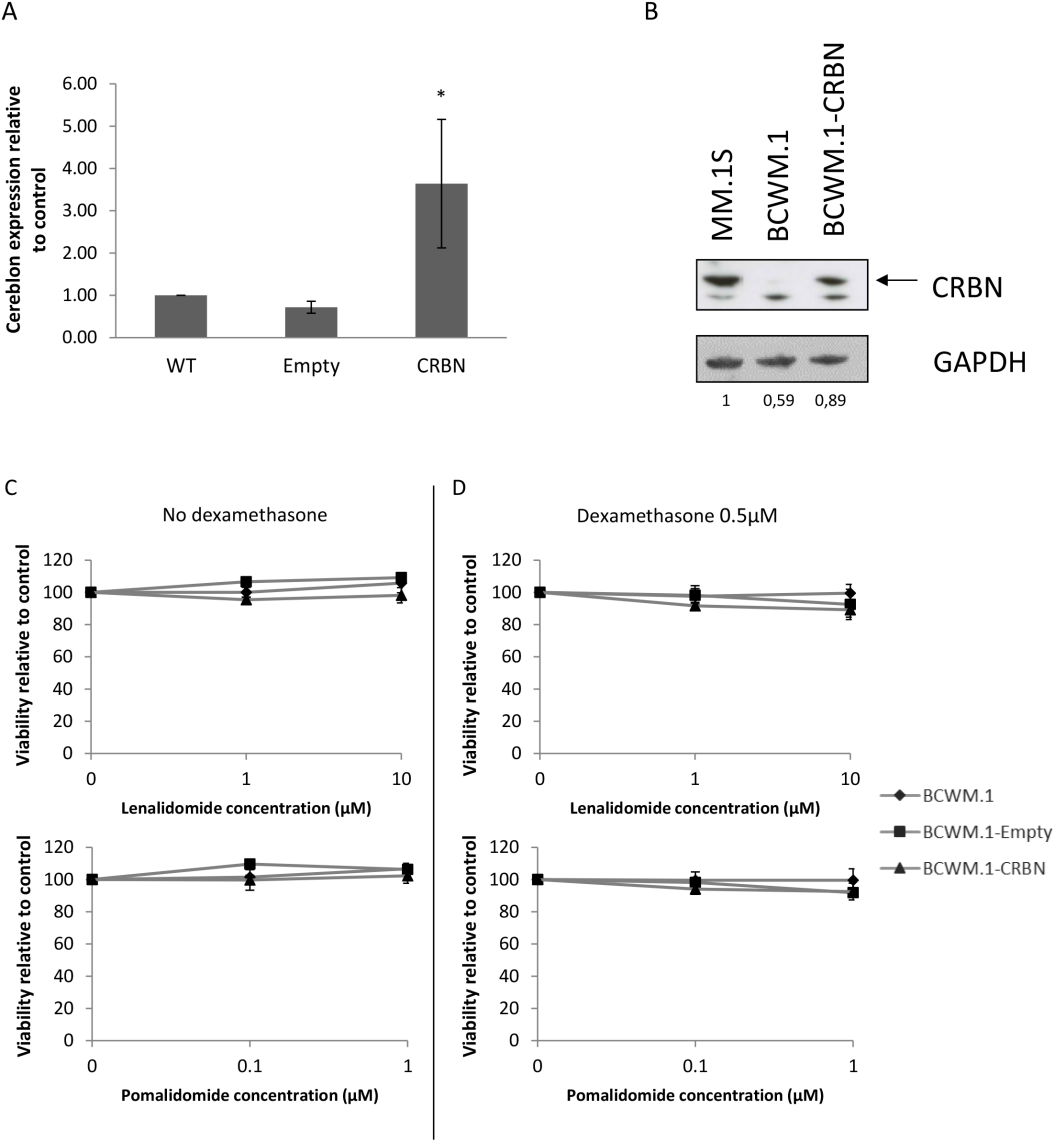
Cereblon is not a key factor in WM cell resistance to lenalidomide and pomalidomide **(A)** qRT-PCR analysis of CRBN mRNA levels in BCWM.1 wild type (WT) cell line, expressing stably CRBN (BCWM.1-CRBN) or the empty vector (BCWM.1-Empty). CRBN is 3.6 times higher after transduction compared to the wild type cell line. ^*^: *p*<0.05 **(B)** Western blot analysis using anti-CRBN mouse antibody showing differences of CRBN expression in BCWM.1 cell lines. MM.1S cells stand as a positive control of CRBN expressing cell line. Band intensities were quantified using ImageQuant TL software, normalized to their respective GAPDH bands and expressed comparatively to MM.1S. **(C** and **D)** Viability (MTS) analysis of BCWM.1 cell line over-expressing or not CRBN after 48 hours exposure to lenalidomide and pomalidomide at the indicated concentrations, with (C) or without (D) 0.5μM of dexamethasone.

In order to characterize the role of CRBN in the resistance of WM cells to lenalidomide and pomalidomide, the BCWM.1 cell line was infected with a lentiviral construct to stably over-express CRBN (Figure 3A and 3B). However no change in cell viability was observed after lenalidomide and pomalidomide treatment in BCWM.1, even when adding dexamethasone, a drug commonly used in combination to IMid compounds in MM to sensitize cells to IMids [14] (Figure 3C and 3D).

Thus, our results suggest that CRBN does not participate in the resistance mechanism to lenalidomide and pomalidomide that occurs in WM.

### IMid compounds downregulate IKZF proteins ikaros and aiolos despite a low CRBN expression

**Figure 4:**
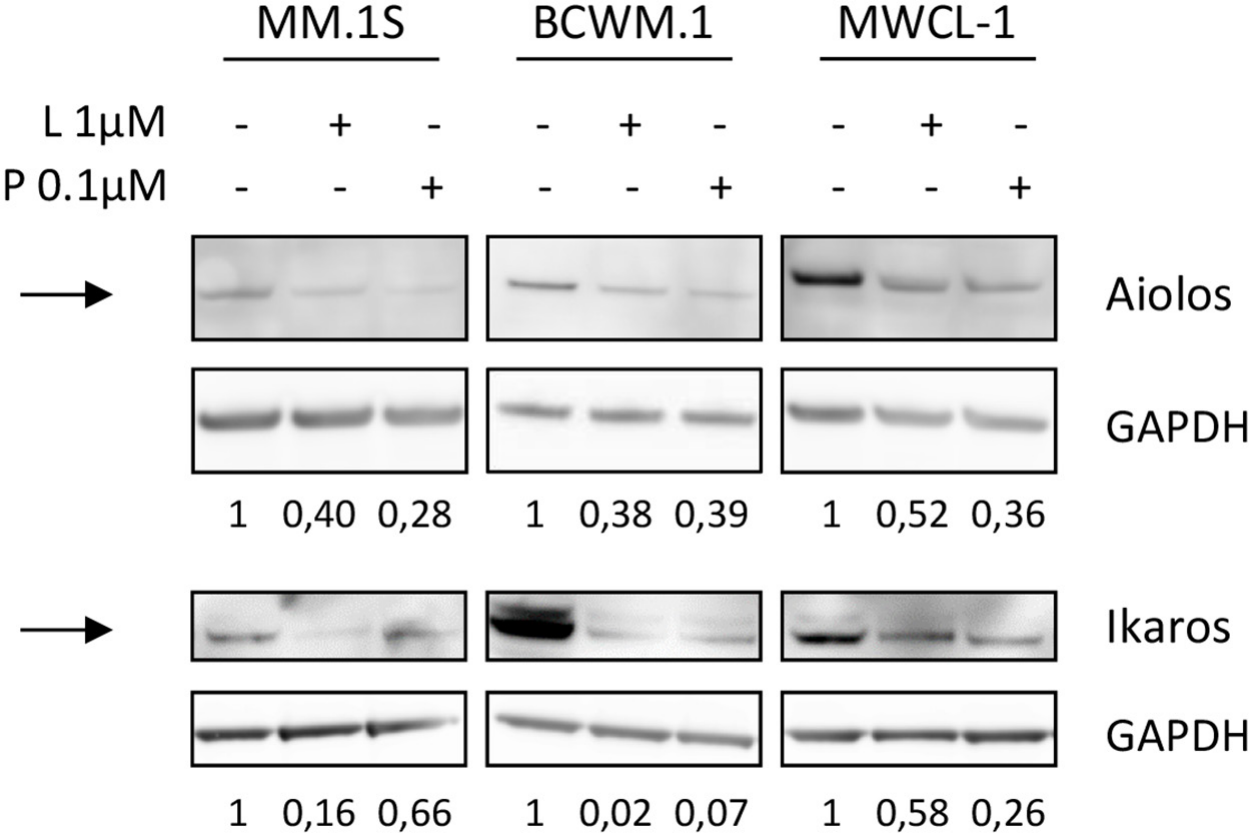
Lenalidomide and pomalidomide are able to modulate Ikaros and Aiolos in WM cell lines, despite low CRBN levels Western blot analysis of Ikaros (IKZF1) and Aiolos (IKZF3) expression in MM and WM cell lines after exposure to lenalidomide and pomalidomide at the indicated concentrations for 48 hours. Band intensities were quantified using ImageQuant TL software, normalized to their respective GAPDH bands and expressed comparatively to the untreated control. Picture is representative of three different experiments. NT: Not Treated; L: Lenalidomide; P: Pomalidomide

As it appeared that CRBN was not involved in the resistance mechanism to IMid compounds in WM, we suspected lenalidomide and pomalidomide were able to modulate the expression of downstream targets that are downregulated as a consequence of the CRBN-IMid compounds binding. Recent studies have identified Ikaros (IKZF3) and Aiolos (IKZF1) as two proteins that are ubiquitinylated and degraded by the proteasome when lenalidomide binds to CRBN [15]. Western blot analysis confirmed that both proteins where degraded after lenalidomide and pomalidomide exposure in WM cell lines (Figure 4).

Hence, the results confirmed that the low level of CRBN was sufficient for lenalidomide and pomalidomide to lead to ubiquitinylation and proteasomal degradation of IKZF1 and IKZF3, despite a lack of impact on cell viability and apoptosis.

### Absence of modulation of IRF4

**Figure 5:**
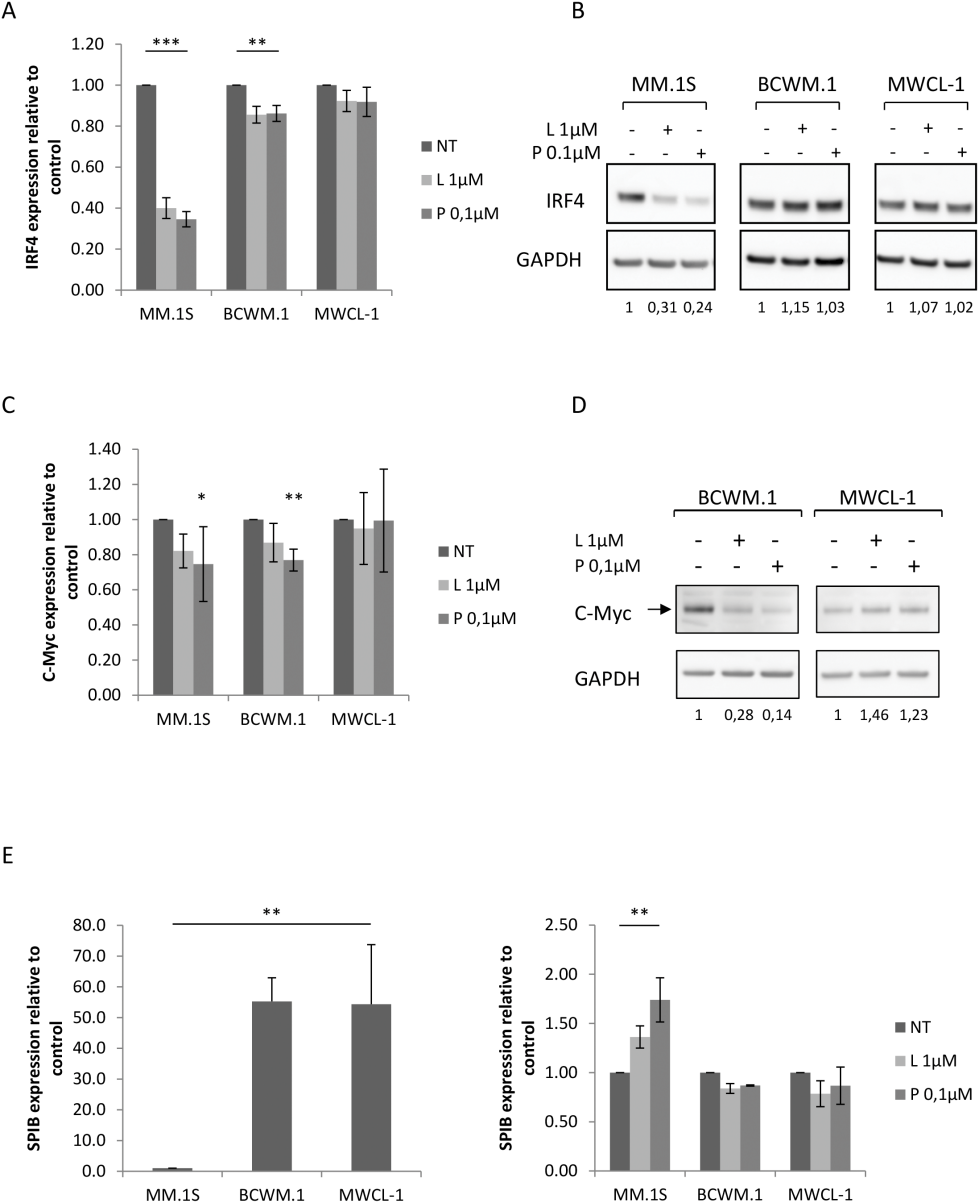
Modulation ofIRF4 regulatory loop in WM cell lines upon lenalidomide and pomalidomide treatment **(A)** Relative mRNA quantitation of IRF4 using qRT-PCR normalized to GAPDH in MM and WM cell lines after 48 hours exposure to lenalidomide and pomalidomide. Upon treatment, mRNA expression is 2.5 and 2.9 times lower respectively in MM.1S cell lines and 1.6 times lower in both conditions in BCWM.1.cell lines. Histograms represent the mean of at least five different experiments +/- SEM. **(B)** Western blot analysis using anti-IRF4 mouse monoclonal antibody showing the decrease of IRF4 expression after 48 hours exposure to lenalidomide and pomalidomide at the indicated concentrations. Band intensities were quantified using ImageQuant TL software, normalized to their respective GAPDH bands and expressed comparatively to the untreated control. Picture is representative of at least three different experiments. **(C)** Relative mRNA quantitation of c-Myc using qRT-PCR normalized to GAPDH in MM and WM cell lines and **(D)** western blot analysis showing a decrease of c-Myc expression in BCWM.1 after 48 hours exposure to lenalidomide and pomalidomide. Band intensities were quantified using ImageQuant TL software, normalized to their respective GAPDH bands and expressed comparatively to the untreated control. Histograms represent the mean of at least three different experiments +/- SD and picture is representative of at least three different experiments. **(E)** Relative mRNA quantitation of SPIB using qRT-PCR normalized to GAPDH in MM and WM cell lines untreated (left) or upon lenalidomide and pomalidomide (right) exposure. Histograms represent the mean of at least three different experiments +/- SD. ^*^: *p*<0.05; ^**^: *p*<0.01; ^***^: *p*<0.001 NT: Not Treated; L: Lenalidomide; P: Pomalidomide.

One of the consequences of IKZF downregulation upon IMid compounds exposure is a decrease of IRF4 expression [16]. Having demonstrated that lenalidomide and pomalidomide were signaling through CRBN and IKZF proteins in WM cells, we investigated their effects on downstream targets. Although there was a slight decrease in IRF4 RNA expression in BCWM.1 subjected to lenalidomide and pomalidomide treatment, no inhibition at the protein level was observed (Figure 5A and 5B).

MYC is known to form an autoregulatory loop with IRF4 during normal B cell activation, and in MM with similar expression pattern [10]. As IRF4 level remains stable in WM cells upon IMid compounds treatment, MYC expression level was assessed after 48 hours exposure to lenalidomide and pomalidomide, and a degradation of MYC was revealed in BCWM.1 cell line (Figure 5C and 5D). This data further encouraged to pursue our understanding of IRF4 central blockage in the mechanism of resistance to IMid compounds in WM.

SPIB is a transcription factor able to interact with IRF4, whose expression is conversely proportional to the interferon regulatory factor [17]. SPIB expression was much higher in WM cells than in MM.1S (Figure 5E), which goes along with previous studies showing an over expression of SPIB in WM CD19+ cells in comparison to healthy donors [18]. Predictably, SPIB expression was boosted by lenalidomide and pomalidomide treatment in MM.1S cells, although no effect was seen on WM cells (Figure 5D).

Taken together, these results further suggest that a resistance mechanism to lenalidomide and pomalidomide occurs at the level of IRF4 or its direct regulation in our model.

### Understanding the deregulation mechanism of IRF4 in WM

As IRF4 level remains non modulated upon IMid compounds treatments in WM despite a functional upstream pathway, further pathways that target IRF4 and could possibly interfere with lenalidomide and pomalidomide activity were investigated in WM.

**Figure 6:**
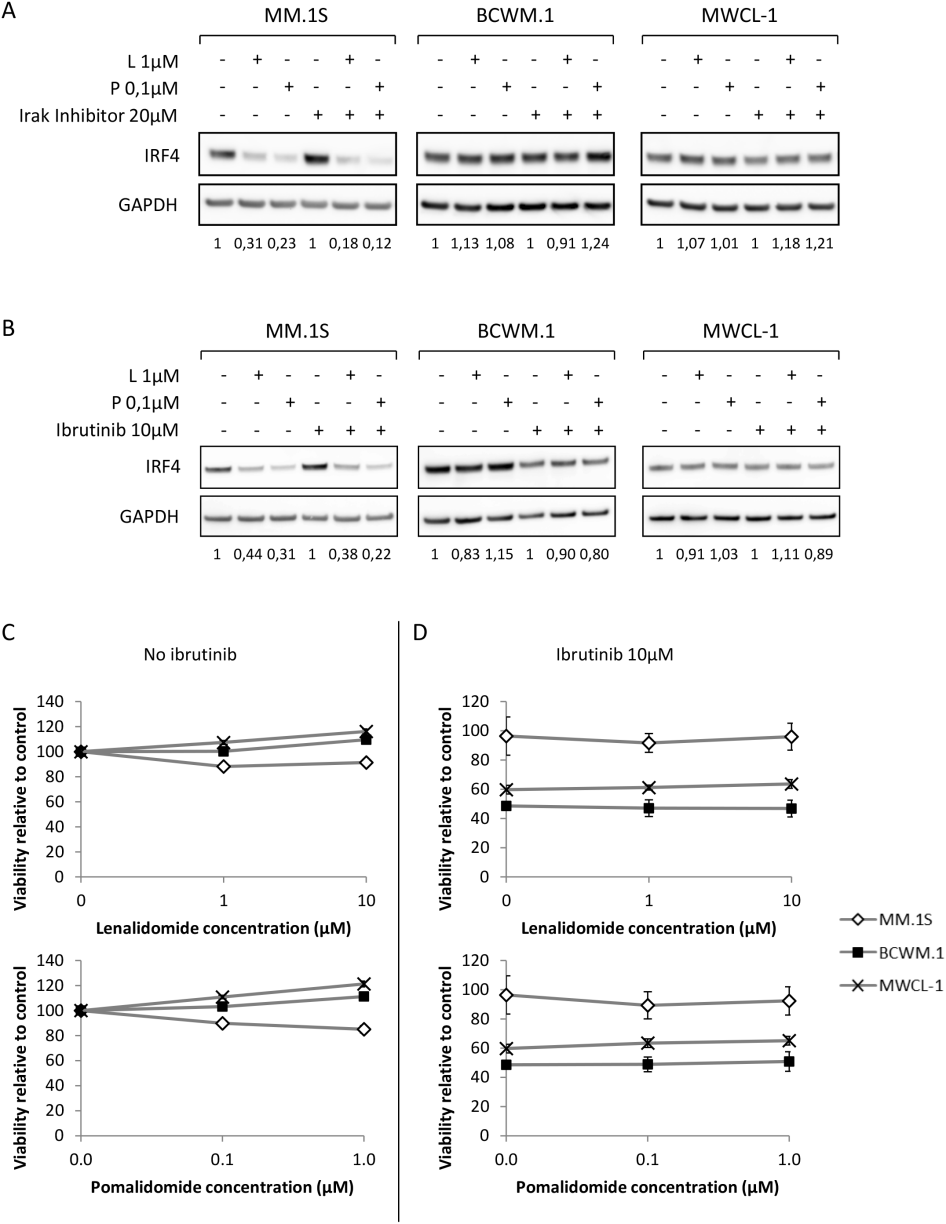
Inhibition of MYD88 and Btk signaling pathways does not sensitize WM cells to lenalidomide and pomalidomide **(A** and **B)** Western blot analysis using anti-IRF4 mouse monoclonal antibody showing IRF4 expression after 48 hours exposure to lenalidomide and pomalidomide at the indicated concentrations with or without Irak inhibitor (A, 20μM) or Ibrutinib (B, 10μM). Band intensities were quantified using ImageQuant TL software, normalized to their respective GAPDH bands and expressed comparatively to the untreated control. Picture is representative of at least three different experiments. **(C** and **D)** Viability (MTS) analysis of the MM or WM cell lines after 48 hours exposure to lenalidomide and pomalidomide at the indicated concentrations, with (C) or without (D) 10μM of Ibrutinib. L: Lenalidomide; P: Pomalidomide.

MYD88^L265P^ mutation induces a constitutive activation of NFKB in WM; thus the impact of its down regulation on IRF4 level was first assessed by inhibiting IRAK1/4, a direct downstream target of MYD88. Our results showed that IRF4 level remained unchanged upon pharmacological inhibition of IRAK1/4 after lenalidomide and pomalidomide exposure (Figure 6A).

Similarly, it was also shown that down regulation of IRF4 could be induced in ABC diffuse large B cell lymphoma after BTK inhibition using ibrutinib. However, BTK inhibition using ibrutinib had no impact on IRF4 level in WM, nor did it increase WM tumor cells sensitivity to lenalidomide and pomalidomide (Figure 6B to 6D).

As a result, we showed that lenalidomide and pomalidomide did not impact IRF4 regulation. Any attempt to potentialize IMids effects or to downregulate IRF4 targeting other pathways failed to decrease its expression in WM tumor cells. Thus, our data confirms that IRF4 regulation is critical to the sensitivity of lenalidomide and pomalidomide to WM.

## DISCUSSION

Despite progress in disease management increasing the median survival of patients, Waldenström's macroglubulinemia is still not a curable disease [4]. Lenalidomide and pomalidomide, representing first-in-class and next generation IMid compounds, respectively, are oral agents that were first developed in treatment of multiple myeloma [19]. As both agents demonstrated an acceptable efficacy and safety profile in treating certain stages of multiple myeloma, they have been further investigated in WM [2]. As we observed a lesser extent of response in WM, we wondered what mechanisms of resistance to lenalidomide and pomalidomide was developed in WM compared to MM.

It has been published that IMid compounds bind to cereblon, a protein part of the E3 ubiquitin ligase complex [20], and trigger a change of CRBN targets [15] leading to an ubiquitinylation and proteasomal degradation of two IKZF proteins, Ikaros (IKZF1) and Aiolos (IKZF3). This downregulation ultimately leads to a decrease of IRF4 expression, a member of the interferon regulatory factor family, a B-lymphocyte specific key transcription factor, often deregulated in MM, and that modulates the expression of several genes involved in cell proliferation and survival [10].

Herein we tried to decipher the mechanisms of action of lenalidomide and pomalidomide in WM, thus possibly understanding the mechanisms of resistance, using two WM-derived cell lines, BCWM.1 and MWCL-1 as model to our study. Interestingly, we were not able to see any action of the IMid compounds on the viability, survival, proliferation or even apoptosis of the WM cells and on CD19 positive BM-derived tumour cells isolated from WM patients.

The first mechanism of resistance to IMid compounds described in MM was a downregulation of CRBN expression in tumour cells [8]. We confirmed that cereblon level was indeed lower in our WM model than in MM, although its overexpression did not restore drug sensitivity. We then looked downstream of CRBN to determine what target was hit upon lenalidomide and pomalidomide treatment, and surprisingly we found that IMids compounds were able to cause IKZF degradation in WM, and to induce a decrease of c-Myc, which interact directly with IRF4. Thus, CRBN expression, although low in WM, is sufficient to efficiently downregulate its below targets. Interestingly, the final downregulation of IRF4 was not observed in WM cell lines upon lenalidomide and pomalidomide treatment, which was independent of dose or time-attempts, or dexamethasone combination. Therefore, we hypothesized IRF4 stable and non-modulated expression was a cause of resistance, and possibly the major mechanism by which resistance occurred to lenalidomide and pomalidomide in WM.

There are various possibilities for IRF4 deregulation in WM. We first studied the presence of genomic alterations directly targeting IRF4 locus, and found notably the absence of mutation on IRF4 in WM. We then sought to understand whether the BCR, and BCR-interacting pathways, known to be activated in WM [6, 21], and to downstream signal through IRF4 [7], would explain the absence of downregulation of IRF4 upon lenalidomide and pomalidomide treatment. Interfering with MYD88 and BTK pathways did not affect IRF4 expression, and was not able to restore lenalidomide or pomalidomide activity in WM. Finally, it is known that IRF4 and SPIB can dimerize [22], and recent studies have shown that SPIB expression was higher in WM lymphoplasmacytic cells than in normal B cells, and hence should lead to a decrease of IRF4 in WM cells [18]. Interestingly, in our study, while lenalidomide and pomalidomide were responsible for an increased level of SPIB in MM cells, no effects were observed in WM. Our data tend to point IRF4 as the pivotal mechanism of resistance to lenalidomide and pomalidomide in WM, which exact cause remains unraveled to date.

As a conclusion, our study depicted the mechanisms of action of lenalidomide and pomalidomide through cereblon binding, and demonstrated a functional CRBN pathway, although baseline CRBN expression was low. We also showed that most downstream targets of lenalidomide and pomalidomide were expectedly affected, but IRF4. Our study proposed therefore a mechanism of resistance to lenalidomide and pomalidomide in WM through deregulation of the expression of IRF4, which cause remains to be unraveled. As no significant effects of lenalidomide or pomalidomide were observed on WM cells, although some patients may respond to treatment, it is suspected there might be indirect mechanisms of action IMid compounds in WM, and it would be interesting to decipher their interaction with the microenvironment and their potential effects on the relationship between tumor and stromal cells [23–25].

## MATERIALS AND METHODS

### Cell culture and reagents

MM cell line MM.1S and WM-derived cell lines BCWM.1 and MWCL-1 were used in this study. BCWM.1 (2010, kind gift from Dr S.P. Treon, Dana-Farber Cancer Institute Boston, MA) and MM.1S (2010, kind gift from Dr S. Rosen, Northwestern University, Chicago, IL) were cultured in RPMI-1640 medium supplemented with 10% fetal bovine serum and 100U/mL of penicillin/streptomycin. MWCL-1 (2010, kind gift from Dr S. Ansell, Mayo Clinic, Rochester, MN) was cultured in Iscove Modified Dulbecco Medium supplemented with 10% fetal bovine serum and 100 U/mL of penicillin/streptomycin. Cells were kept at 37°C, 5% CO_2_ in humid atmosphere. Phenotypes were tested using flow cytometry at cell line delivery and mycoplasma testing was done using MycoAlert™ Mycoplasma Detection kit (Lonza, Basel, Switzerland) after vial thawing, every two months.

CD19+ cells from WM patients were isolated as described [6] and cultured in RPMI-1640 supplemented with 10% fetal bovine serum and 100U/mL of penicillin/streptomycin. Informed consent was obtained prior to research sampling. Lenalidomide and pomalidomide were provided by Celgene. All drugs tested were purchased from Selleck chemicals (Houston, TX) apart from the IRAK 1/4 inhibitor (Merck Millipore, Darmstadt, Germany).

### Lentiviral constructs

Lentiviral constructs expressing resistance to puromycin used to generate stable CRBN expressing BCWM.1 cell line (BCWM.1-CRBN) and empty vector (BCWM.1-Empty) were purchased from Applied Biological Material (ABM) Inc. (Richmond, BC). BCWM.1 cell line was exposed to lentiviral particles for 6 hours. Two days after the infection, selection of the infected cells was done by adding puromycin to the culture medium at a concentration of 1μg/mL.

### Western blotting, apoptosis and viability assays

Cell viability was assessed by MTS reagent (3-4,5-dimethylthiazol-2yl)-5-(3-carboxy-methoxyphenyl)-2-(4sulfophenyl)-2H-tetrazolium, inner salt) following the manufacturer's instruction (Promega, Madison, WI). Apoptosis was measured using Annexin V-FITC kit (Beckman Coulter Inc., Pasadena, CA) according to the manufacturer protocol. Staining was detected and analyzed on a CyAn ADP analyzer flow cytometer (Beckman Coulter Inc.).

For the western blot analysis, whole cell proteic lysates were subjected to 4-12% sodium dodecyl sulfate polyacrylamide gel electrophoresis, transferred to nitrocellulose membrane (Thermo Fisher Scientific Inc., Waltham, MA) and incubated with antibodies directed against CRBN (kind gift from Dr H. Handa, Tokyo Medical University, Toko, Japan), IRF4, GAPDH (Santa Cruz Biotechnology, Santa Cruz, CA), β-actin (Sigma-Aldrich Co., St. Louis, MO), Ikaros and Aiolos (Cell Signaling, Beverly, MA). Blots were then visualized using the Amersham ECL Prime Western Blotting System on a Las 4000 biomolecular imager (GE Healthcare Europe GmbH, Velizy-Villacoublay, France). Quantification was done using ImageQuant TL software (GE Healthcare, Chicago, IL).

### Quantitative RT-PCR

Total RNA was purified (Nucleospin RNA isolation kit, Macherey-Nagel GmbH, Düren, Germany) and then reverse transcribed into cDNA using the Superscript^®^ VILO cDNA synthesis kit (Thermo Fisher Scientific Inc.) following the manufacturer's instructions. Next, the generated cDNA was subjected to quantitative PCR using the Taqman^®^ Universal Master Mix and the following Gene Expression assays: CRBN (Hs00372271_m1), IRF4 (Hs01056533_m1), Ikaros (IKZF1, Hs00958474_m1), Aiolos (KZF3, Hs00232635_m1), containing specific Taqman^®^ probes and primers (Thermo Scientific Inc.). The quantity of product was normalized to glyceraldehyde-3-phosphate deshydrogenase (GAPDH, Hs03929097_g1) as the endogenous housekeeping gene. Fold change of gene expression was calculated using the comparative Ct method (2^-ΔΔCt^).

### Statistical analysis

Statistical differences between drug-treated and control conditions were determined using the Student's t-test. *P* values <0.05 were considered statistically significant.
